# Innate or Acquired? – Disentangling Number Sense and Early Number Competencies

**DOI:** 10.3389/fpsyg.2018.00571

**Published:** 2018-04-19

**Authors:** Julia Siemann, Franz Petermann

**Affiliations:** ^1^Department of Medical Psychology and Medical Sociology, University Medical Center Schleswig-Holstein, Kiel, Germany; ^2^Center for Clinical Psychology and Rehabilitation, University of Bremen, Bremen, Germany

**Keywords:** dyscalculia, domain specificity, innate number sense, subtypes, early number competence

## Abstract

The clinical profile termed developmental dyscalculia (DD) is a fundamental disability affecting children already prior to arithmetic schooling, but the formal diagnosis is often only made during school years. The manifold associated deficits depend on age, education, developmental stage, and task requirements. Despite a large body of studies, the underlying mechanisms remain dubious. Conflicting findings have stimulated opposing theories, each presenting enough empirical support to remain a possible alternative. A so far unresolved question concerns the debate whether a putative innate number sense is required for successful arithmetic achievement as opposed to a pure reliance on domain-general cognitive factors. Here, we outline that the controversy arises due to ambiguous conceptualizations of the number sense. It is common practice to use early number competence as a proxy for innate magnitude processing, even though it requires knowledge of the number system. Therefore, such findings reflect the degree to which quantity is successfully transferred into symbols rather than informing about quantity representation *per se*. To solve this issue, we propose a three-factor account and incorporate it into the partly overlapping suggestions in the literature regarding the etiology of different DD profiles. The proposed view on DD is especially beneficial because it is applicable to more complex theories identifying a conglomerate of deficits as underlying cause of DD.

## Scope

In the present selective review, we discuss normal and abnormal arithmetic development. We present current positions on the central questions of:

(a) precursors for successful mathematical education
(b) risk factors for low math performance
(c) relative contributions of domain-specific and domain-general factors
(d) heterogeneity of dyscalculia symptoms.

As a starting point, we will outline the current knowledge on arithmetic acquisition separately for domain-general and domain-specific contributing factors. Based on these findings, we will then explain the key deviations from the regular developmental path present in children with dyscalculia according to the literature. For this purpose, typical findings on healthy children with regard to contributions of domain-general as well as domain-specific factors are outlined. Afterward, these are delineated from maladaptive mathematical development.

Next, we will turn to the central question of heterogeneity in developmental dyscalculia (DD). At present, there are still diverse suggested key abnormalities in the literature based on contradictory study results. From this, we will turn to an associated problem: despite the general agreement that there are subtypes of math difficulties, there is an apparent gap with respect to cognitive processes. Here, we wish to put forward that a finer distinction between innate number sense and early number competence helps in disentangling studies contradicting each other. For that purpose, we introduce a three-factor account that is based on past findings and extends previous models. We complement the above by bringing forward several potential reasons leading to different concepts of DD. Finally, we reconcile these seemingly incompatible positions by suggesting how future studies could benefit from our conception of arithmetic development and DD.

## Healthy Math Development: Interactions Between Domain-Specific and Domain-General Factors

Before turning to DD and its possible causes, we briefly describe how healthy math development proceeds, because theories on DD are necessarily grounded on this background knowledge. The mammalian brain seems to be equipped with an innate and preverbal ability to differentiate between quantities (e.g., Kucian and von Aster, 2015), the so-called “number sense” (Dehaene and Cohen, 1997). Humans (and other species) can learn to associate this system with symbolic number representations. The latter mechanism apparently evolves in parallel (Hyde, 2011) or hierarchically (von Aster and Shalev, 2007) into the exact and automatic recognition of small amounts of up to four or five items (“subitizing,” e.g., Henik et al., 2012; see Piazza, 2010 postulating a precursor object tracking system) and the approximate discrimination between larger quantities [“approximate number system” (ANS), e.g., Feigenson et al., 2004). Similar theories postulate a “one system view” of number representation (Hyde, 2011). Subitizing and ANS thus refer to complementary mechanisms to differentiate small (exact) or large (approximate) numbers, i.e., distinct aspects of the number sense. In concert, they enable the comprehension of cardinality and ordinality (number concept and placement principles, Rapin, 2016). These mathematical principles are crucial for arithmetic and serve as early diagnostic markers (Gray and Reeve, 2014).

Innate basic abilities and acquired general skills both contribute to math development. Geary (2007) discriminates between so-called primary vs. secondary precursors to account for abilities we are biologically endowed with (biologically primary) from skills shaped by environmental influences (biologically secondary). In the following, we will use the more general terms of domain-specific vs. domain-general (e.g., Karmiloff-Smith, 2015). Notably, some studies treat acquired numerical operations (e.g., calculation and arithmetic) as domain-specific (see conceptualization of Gersten and Chard, 1999), and the National Mathematics Advisory Panel even defines number sense as the understanding of the basic concept of numbers (precise representation of small and approximation of large numbers, counting skills, and simple numerical operations; National Mathematics Advisory Panel, 2008) rather than of magnitude *per se*. This example shows that skills related to early number competencies are taken as proxies for innate number abilities. To disambiguate these distinct concepts (early number competence and magnitude processing), we conceive of number sense as a pre-educational ability (following Berch, 2005) such as magnitude processing and estimation abilities. This differentiation is crucial when interpreting contradictory empirical findings and constitutes the starting point of our three-factor account. For that reason, it is important to consider both contributing factors (primary and secondary), as outlined below for healthy arithmetic development.

### Domain-Specific Abilities

There are several theoretical considerations on math development. For example, von Aster and Shalev (2007) suggest a four-step-model of numerical development from discrete numerosity processing to abstract concepts of magnitude. Therein, domain-specific subitizing is a precursor of counting and subsequently for associating explicit symbolic representations (number words and Arabic digits) with the implicit number sense, culminating in the acquisition of a mental representation of numbers that is spatially organized on a mental number line. The model is based on the triple-code model of number processing (Dehaene et al., 2003) and sketches key brain structures for each developmental stage. Accordingly, there is empirical evidence for brain maturation processes during math learning with regard to structure (Zamarian et al., 2009), function (Rapin, 2016), and connectivity (Moeller et al., 2015). Yet, being explicitly formulated in the context of abnormal mathematical development, the four-step model may not cover the entire spectrum of developmental mechanisms in healthy children. More comprehensive models such as LeFevre et al.’s (2010) three-pathway model commonly schedule three precursors for math development, consisting of domain-specific quantity representation (including subitizing) and domain-general linguistic skills as well as variable indices of spatial processing (see Krajewski and Schneider, 2009; Cirino, 2011, for similar approaches).

These models incorporate the domain-specific number sense in distinct ways. Competing theories suggest either that ANS and acquired mathematical skills depend on common domain-general cognitive operations (Park and Brannon, 2014) or that their neuronal representations directly overlap (Lindskog et al., 2014), yet neither accounts for the diverse findings on the relation between ANS and math so far (see Hyde et al., 2016). This may result from the way that number sense and early number competence are defined and especially whether ANS is assigned to one (e.g., Szücs and Myers, 2017) or the other (e.g., Jordan et al., 2007).

While von Aster and Shalev (2007) define subitizing as an innate ability that is required for counting (i.e., number sense as we define it here), LeFevre et al.’s (2010) model treats magnitude processing as being synonymous with early numeracy knowledge. Moreover, in empirical studies, there is a tendency to collapse over these competencies (e.g., Powell and Fuchs, 2012). We believe that discrepant findings in the literature are contingent upon these different conceptualizations. Correspondingly, when operationalizing ANS as a proxy for number sense, only moderate levels of correlation with mathematical skills were found in adults (Chen and Li, 2014; Fazio et al., 2014) and in infants (Bonny and Lourenco, 2013) when measured concordantly (cross-sectional studies). Longitudinal studies further point to a genuine causal involvement, as expertise in ANS predicts later math growth (Libertus et al., 2013a). However, this relation decreases with age (Bonny and Lourenco, 2013; Fazio et al., 2014), hinting at a mediating role of the ANS. Thus, Libertus et al. (2013a) found the ANS to work indirectly via early number competencies, which then predict later math achievement. Accordingly, ANS acuity impacts on early number competence but not formal math skills (Libertus et al., 2013b), and the predictive impact of symbolic quantity measures exceeds that of non-symbolic scores (Sasanguie et al., 2012). This may also apply to evidence in the literature that math growth and ANS are apparently uncorrelated (Sasanguie et al., 2013; Szücs et al., 2014). Accordingly, studies operationalizing domain-specific quantity processing via early number competence report a stronger correlation with later mathematical abilities (Jordan et al., 2007; Chu and Geary, 2015). Indeed, Sasanguie et al. (2015) suggest a binary magnitude system with separate modules for exact and approximate quantities. Likewise, Kucian and Kaufmann’s (2009) model of number representation for healthy math development explicitly conceptualizes the increasing overlap between different quantity representations with age. The model is in line with the discussed findings as number becomes an abstract concept detached from concrete number representations. Such novel considerations are extensively discussed in a recent meta-analysis taking into account developmental shifts as well as different ANS operationalization measures (Schneider et al., 2017).

In sum, domain-specific magnitude processing (i.e., number sense) is at the heart of most contemporary models seeking to explain developmental trajectories of mathematical processing. Unfortunately, it remains a matter of debate whether magnitude processing is indeed abstract with a dedicated domain-specific module (see the discussion in Cohen Kadosh and Walsh, 2009). Novel conceptualizations of arithmetic development are in need, and existing accounts lack a comprehensive conceptualization that accounts for numerous discrepant findings in the literature (LeFevre, 2016). The matter is further complicated by the diverse influences of secondary precursors that are not easily disambiguated from potential primary causes (see Traeff et al., 2017 on this matter). Moreover, their relative contributions seem to be accompanied by an age-dependent shift. Evidence on domain-general skills will be addressed in the following.

### Domain-General Skills

While the previous paragraph stresses the importance of domain-specific precursors for healthy math development, other studies are devoted to the role of domain-general factors. Several early general skills predict later school math longitudinally, including visuospatial properties (Lauer and Lourenco, 2016; Verdine et al., 2017), intelligence (Dumontheil and Klingberg, 2012; Hornung et al., 2014), linguistic skills (Praet et al., 2013; Zhang et al., 2014), executive control (Bull et al., 2008; Clark et al., 2013), and working memory (LeFevre et al., 2013; Bailey et al., 2014; but see Fuchs et al., 2006). While working memory span has often been considered essential to math skill levels, this seems to be content-specific. In fact, visuospatial rather than verbal WM skills correlate with math achievement in healthy populations (Clearman et al., 2017), whereas patients with DD show stronger correlations with verbal WM (Mammarella et al., 2013). Accordingly, Szücs (2016) identified type of WM impairment (verbal and visuospatial) as contributing to the specific profile of mathematic problems in DD patients. Moreover, the correlation between WM and math may be stronger in children with low number sense capabilities than healthy controls. Therefore, differentiation between control groups and children with DD is essential when examining domain-general factors. Thus, Szücs et al. (2014) found no correlation between WM and math performance in healthy children. A possible explanation is given by the development of an arithmetic fact memory. Healthy individuals may be able to use their number sense to develop early number competencies (i.e., connections between magnitude and numbers, basic arithmetic principles, etc.) as a basis for an arithmetic fact memory. By contrast, children with DD cannot profit from such automated processes, rather relying on immature mental calculation strategies such as counting. These in turn draw heavily on verbal WM capacities (Alloway et al., 2006), probably leading to a stronger connection between arithmetic and WM. Correspondingly, WM seems to be especially important for more sophisticated math operations such as subtraction (Caviola et al., 2014). Finger counting may serve as a compensatory function to offload WM (Crollen et al., 2011) and is frequently observed in DD (Attout and Majerus, 2015). Nonetheless, domain-specific abilities still contribute to later math outcomes over and above general cognitive influences. Thus, elementary and middle school addition both correlate with early number comparison skills irrespective of working memory, visuospatial skills, linguistic performance, and IQ (Bailey et al., 2014). Moreover, early enumeration capacity uniquely accounts for arithmetic achievement when controlling for working memory and executive functions (Gray and Reeve, 2014). A recent meta-analysis further suggests that early number competence but not WM predicts calculation performance in at-risk children (Peng et al., 2016a). In addition, while math training programs were found to have the largest effects on early number competence, improving domain-general cognitive skills does not seem to transfer to enhanced mathematical achievement (see Raghubar and Barnes, 2017).

Emphasizing the developmental nature of healthy mathematical acquisition may help to reconcile these findings, as both factors (domain-specific and domain-general) apparently contribute to distinct aspects during math growth (Hornung et al., 2014). As a synopsis of the presented considerations, there are many influential factors on arithmetic acquisition: age of participants may determine whether domain-specific or domain-general performance predominantly correlates with math achievement; test format (verbal and visual) may lead to different results especially with respect to correlations with WM; and sample type (healthy and DD) seems to lead to different correlations due to distinct strategies. With this knowledge in mind, the following paragraphs will point to domain-specific and domain-general abnormalities during mathematical development that may cause the disorder labeled DD.

## Developmental Dyscalculia

### Nomenclature

So far, there is no unitary expression for DD, as it is a complex disorder that may be associated with diverse problems including low math performance, low counting skills, weak arithmetic, struggles with calculation, or inabilities in understanding mathematical procedures. Accordingly, synonyms such as “persistent mathematical difficulties” (Morgan et al., 2016), “mathematics learning disability” (Murphy et al., 2007), or “mathematical difficulties” (Schwenk et al., 2017) may be used to delineate profound (maybe innate) magnitude processing from presumably acquired problem with arithmetic (see Morgan et al., 2016). This diversity of expressions for the same basic collection of symptom already indicates that there is neither a unified concept of mathematical disorders nor a consistent etiological explanation thereof. The term “mathematics learning disability” stresses the role of domain-general operations in learning mathematical proficiency and is predominantly applied by opponents of an innate number sense problem (e.g., Rousselle and Noël, 2007). By contrast, “mathematical difficulties” seem to represent a severity-based expression, leaving open the possibility for an innate as well as an acquired etiology.

The current version of the ICD-10 [International Classification of Diseases; World Health Organization [WHO], 1992] classifies dyscalculia among the pervasive and specific developmental disorders (chapter F8) as a specific developmental disorder of scholastic skills (sub-chapter F81) as a mathematical disorder (F81.2) with no further specification. The criteria demand a discrepancy between a child’s intelligence level and a standardized math test score as well as adequate mathematical educational circumstances. By contrast, in the latest version of the Diagnostic and Statistical Manual of Mental Disorders (5th ed.; DSM–5; American Psychiatric Association, 2013), DD is listed as *specific learning disorder with impairment in mathematics* (315.1) and may be grounded on problems with the number sense as well as with arithmetic fact retrieval, calculation, or math reasoning. The release of the revised version ICD-11 is still pending. It is to be expected that similar alterations with respect to intelligence level and mathematical scores will be put forward.

In the following, we will summarize both well-established and more recent positions on pathological math development separately for potential domain-specific and domain-general precursor abilities and integrate the gathered knowledge into a more precise view on DD.

### Presumable “Domain-Specific” Symptoms Associated With DD

Developmental dyscalculia in children is characterized by profound difficulties with various fields of mathematics, including counting principles, transcoding between number digits and number words, comprehension of number syntax, numerical fact knowledge, and fact retrieval (Jordan et al., 2003). A deficient *number sense* is most frequently related to DD (according to Mazzocco and Thompson, 2005), and correlations between ANS acuity and math proficiency apparently exist prior to mathematical education (Mazzocco et al., 2011; Libertus et al., 2013a) and ANS has a predictive role for math performance in young children (Wong et al., 2017). Analogous to evidence for healthy math development (e.g., Bailey et al., 2014), numerical competence (commonly considered to be domain-specific) of at-risk children uniquely predicts math performance during elementary school even when controlling for domain-general skills (Peng et al., 2016b). By contrast, in adults, DD primarily reflects domain-general fact retrieval deficits and weak phonological processing via an impaired association between both (De Smedt and Boets, 2010).

Immature *counting* strategies in DD may be causally related to deficient fact knowledge by hindering the build-up of associations between arithmetic operations and solutions (Geary et al., 2012). Similar findings highlight the importance of progressing from procedure-based counting to memory-based *fact retrieval* (De Visscher and Noël, 2016). Thus, patients with DD are hypersensitive to interference from neighbor problems in multiplication, posing an indirect negative effect by preventing the successful storage of symbol-response associations in long-term memory (De Visscher and Noël, 2016). Still, while children with DD are particularly impaired in grasping the principle of cardinality during counting (Rapin, 2016), which is also predictive of counting in healthy arithmetic development (Moore et al., 2016), the same skill seems to play only a minor role in healthy children. They likewise fail to comprehend this principle despite otherwise healthy mathematical development (Kamawar et al., 2010). Kuhn et al. (2016) infer that DD essentially reflects a deficit of *specific precursor* abilities that healthy infants are endowed with even before learning arithmetic or calculation (i.e., estimation, enumeration, and transcoding). Longitudinal studies accordingly show that basic quantity-based abilities including number naming, counting, and estimation are stable predictors of arithmetic proficiency during the transition from preschool to kindergarten (VanDerHeyden et al., 2006), elementary school (Methe et al., 2008; Lembke and Foegen, 2009), and high school (Siegler et al., 2012), independent of general intelligence levels (Locuniak and Jordan, 2008). It is therefore likely that quantity processing enables more sophisticated mathematical manipulations. However, a recent meta-analysis found symbolic rather than non-symbolic quantity processing measures to be related to low mathematical performance in children with DD (Schwenk et al., 2017). This is in line with the relative contributions of the number sense and of early number competence for healthy arithmetic skills discussed above. The shift from non-symbolic ANS to basic symbolic skills observed in healthy children seems to be aberrant in DD. It is therefore essential to consider supportive skills in contributing to abnormal mathematical development as well. To meet this demand, the following section is devoted to domain-general skills in the context of low mathematical abilities.

### Domain-General Deficits in DD

Apart from poor numerical abilities, low math performance may also be grounded on malfunctioning supportive cognitive operations. Longitudinal and cross-sectional studies repeatedly identified performance differences between children with DD and healthy controls in attention (Ashkenazi et al., 2009; Swanson, 2011), executive functions (especially inhibitory control; Swanson, 2012; Szücs et al., 2013), linguistic skills (as mediator, see Jordan et al., 2015), intelligence (Geary et al., 2008; but see Alloway, 2009), general processing speed (Geary, 2010; Namkung and Fuchs, 2016), and visuospatial processing (Hanich et al., 2001), especially with respect to short-term (Andersson, 2010) or working memory (Ashkenazi et al., 2013; Bugden and Ansari, 2015). Yet evidence on their individual relative contributions is inconclusive, and only few studies systematically explored single cognitive factors in the context of DD (Morgan et al., 2016).

As described initially, diverse skills such as language (Zhang et al., 2014), attention (Morgan et al., 2016), and intelligence (Geary and Moore, 2016) contribute to sophisticated (i.e., healthy) math knowledge. However, findings on healthy mathematical development have limited values for the etiology of DD. Especially with regard to procedural knowledge, evidence on DD is still in need. To our knowledge, only three studies have specifically addressed calculation development (reflecting procedural knowledge) in DD in comparison with healthy controls using longitudinal data so far (according to Peng et al., 2016b). Of these, Alloway (2009) found a correlation between working memory (but not intelligence) and calculation, but without controlling for other variables such as verbal skills or executive functions. In a broader approach, Namkung and Fuchs (2016) found processing speed and attention to predict later calculation expertise in DD, whereas language skills did not explain additional variance. The only study we identified (Peng et al., 2016b) that addressed this matter with data from elementary school (i.e., early math development) suggests that

(a) processing speed (domain-general) and early number competence predict mathematical growth in DD
(b) early number competence mediates the degree to which DD persists at the end of elementary school
(c) children with DD and comorbid reading disorder compensate their deficits using early number competence rather than domain-general skills.

Peng et al. (2016b) also report independence between linguistic skills and whole number math in DD. In contrast to this finding, others suggest that linguistic deficits may be linked with DD in school-aged children (Fuchs et al., 2006), paralleling the relation between language and healthy math development. Consequently, assuming a direct link between reading disorder and DD – potentially with a causal relationship – is tempting. Yet evidence in this field is contradictory, as longitudinal positive correlations between both clinical samples (Jordan et al., 2002) stand against contrary findings (Andersson, 2010). Moreover, a potentially underlying impact of linguistic deficits on arithmetic fact retrieval in DD (according to Simmons and Singleton, 2008) could not be substantiated unequivocally (e.g., Geary et al., 2012).

This list of deficits associated with DD is far from complete and demonstrates how intricate it is to interpret low math performance in the broader context of mathematical development, especially when compared to healthy children and adult mathematics. This discrepancy stresses the importance of a multifactorial approach in the etiology of DD. In the next sections, we will outline opposing viewpoints in the literature with regard to the question whether abnormal mathematical development is necessarily caused by an underdeveloped number sense and will show how a finer distinction between different precursors for distinct DD subtypes can reconcile ambiguous study results.

### Heterogeneity in DD

In light of the above findings, it appears that the precursors for successful math development differ from those abilities frequently impaired in DD. Healthy math skills are likely to be continuously distributed, whereas DD constitutes profound deficits distinct from the low end of this continuum (see Desoete et al., 2012). Indeed, whereas healthy math performance scores are highly variable from preschool to elementary school (Geary et al., 2000) and persistent interindividual differences only emerge at grade 2 (Jordan et al., 2003), possibly based on changing strategies (Aunola et al., 2013), DD is stable over time (Andersson, 2010). The most obvious demarcation between healthy math development and DD is evident when considering that low initial numerical competence in elementary school is often not clinically significant in follow-up tests anymore (Desoete et al., 2012). Obviously, manifold reasons can account for weak performance in math tests and need to be identified before erroneously diagnosing DD (see Kaufmann et al., 2013 for a discussion). Nonetheless, growth of mathematical proficiency depends decisively on an individual’s initial numerical competence even before school (Jordan et al., 2009), suggesting that mathematical cognition may be less unitary than conceptualized in many studies (see Dowker, 2008 on individual differences).

Unfortunately, research so far lacks insight into early developmental influences of deficient precursors specifically in DD, because most studies either address later developmental stages (elementary school) or apply cross-sectional study designs impeding a proper analysis of causal influences (see Peng et al., 2016b). However, children’s age represents a major contributing factor to the causes of DD and correlations with other skills. Thus, certain precursors are only transiently related to DD (Knievel et al., 2011), and despite the fundamental role of the so-called number sense, domain-general influences must be taken into account to differentiate between DD profiles (Szücs, 2016). For example, domain-general visuospatial (Passolunghi et al., 2008) and decoding skills (Peng et al., 2016b) contribute to arithmetic acquisition but not later math proficiency. Likewise, the predictive role of the ANS decreases with age (Szkudlarek and Brannon, 2017), and while early number competency emerge as an initial predictor, domain-general skills gain more importance through arithmetic development (Geary et al., 2017). Failing to control for such transitory effects may in turn result in contradicting findings such that children with DD can demonstrate age-adequate domain-specific number processing competence. Accordingly, children diagnosed with DD in grade 2 showed comparable number processing profiles compared with a control group in grade 4 (Landerl and Kölle, 2009). Data from Fuchs et al. (2010) suggest that it is crucial to be cautious about the manifest variables chosen to operationalize DD. In that study, only mathematical *word problem* skills varied with basic numerical abilities, whereas *calculation* performance did not correlate with other domain-general or domain-specific variables (Fuchs et al., 2010). Neuroimaging findings suggest that the bilateral inferior parietal lobule executes domain-specific magnitude processing (Dehaene et al., 2003) and exhibits disparities in DD (Mórocz et al., 2012). However, the same structure is also engaged in domain-general skills that contribute to arithmetic, like working memory (Dumontheil and Klingberg, 2012), attention (Vandenberghe et al., 2012), and spatial processing (Yang et al., 2012). This emphasizes the diversity of DD profiles and leads to an important question raised in the literature about the etiology of distinct DD subtypes (see Andersson and Östergren, 2012 for a review).

### Etiology and Subtypes

In the last decades of DD research, four distinct classes of theories have emerged (according to Castro-Canizares et al., 2009). The first suggests that a domain-specific number sense deficit underlies DD, either for approximate and analogous quantities (number sense deficit, Wilson and Dehaene, 2007) or for exact and discrete representations thereof (defective number module, Butterworth, 2005a).

Alternatively, DD may stem from poor access to quantity information, i.e., an aberrant communication between brain regions devoted to magnitude and its symbolic representation (access deficit, Rousselle and Noël, 2007).

The third class proposes a generalized magnitude system in the brain (comprising both exact and abstract quantities and extending to numbers, time, and space) that is malfunctioning in persons with DD (a theory of magnitude, Cohen Kadosh et al., 2008).

Finally, a forth class of theories identifies a causal relation between mal-efficient domain-general factors and DD symptoms (cognitive deficits, Geary et al., 2007).

At the interface of these accounts, double deficit theories assume that deficits of multiple neuropsychological abilities contribute to learning disabilities in general (Wolf and Bowers, 1999). However, there are no consistent findings in the literature. Accordingly, whereas rapid automatized naming of digits and phonological awareness did not predict DD in a previous study (Heikkilä et al., 2016), another study found similar operations (processing speed and verbal comprehension) to correlate with DD symptoms (Willcutt et al., 2013). Moreover, low performance in number comparison tasks is inconclusive with regard to the underlying deficit, because while this problem may stem from a defective innate number processing system (Butterworth, 2005b), an alternative explanation is a deficit in accessing this module (Rousselle and Noël, 2007). Instead, distance and problem size effects may be more informative, as they typically alleviate with development (Holloway and Ansari, 2008) and may be underdeveloped in DD (Skagerlund and Traff, 2016). In addition, there are hardly any physiological findings to support the domain-specific theory on DD (Szücs, 2016). In fact, there is evidence that both domain-general skills and domain-specific abilities represent superordinate predictors of DD (Toll et al., 2016) that are sensitive to training programs (Kuhn and Holling, 2014). Possible reasons may be a potential dependency of number sense performance on WM during early arithmetic development (Vandervert, 2017) or the fact that tests of ANS (representing the number sense) often cannot disentangle perceptual factors drawing on WM from actual numerical skills (Bugden and Ansari, 2015).

The multitude of DD profiles may actually be grounded on separate (and potentially overlapping) etiologies (e.g., Kucian and von Aster, 2015; Skagerlund and Traff, 2016) reflected at first sight in common deficits in arithmetic performance. Thus, the above classes of hypotheses potentially apply to distinct DD phenotypes and consequently to different underlying causes: whereas a “defective module” (Butterworth, 2005a) or deficient “number sense” (Wilson and Dehaene, 2007) implies that abnormal mathematical development results from an immature magnitude representation; the latter is intact according to the “access deficit” theory (Noël and Rousselle, 2011), which centers on problems retrieving numerosity from symbolic representations (Rousselle and Noël, 2007). Therefore, distinct theories can co-exist and need not be mutually exclusive when more closely investigating the underlying deficits and their operationalization. In the following, we will show that a finer separation of domain-specific deficits dissolves several related issues in DD research.

### A Novel Concept of DD Typology

**FIGURE 1:**
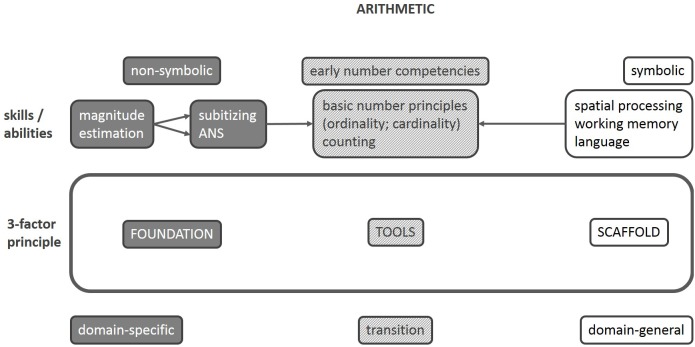
Schematic illustration of the proposed three-factor model. Putative innate skills as well as supporting abilities both contribute to the acquisition of early number competencies required for successful arithmetic.

We suggest that properly characterizing arithmetic development and DD require three factors – as opposed to two in the literature (see **Figure 1**). Factors 1 and 2 have previously been described. The domain-specific number sense (F1) likely represents the *foundation* on which arithmetic development rests. During formal math education, various domain-general skills (F2) assist in linking abstract numerosity with symbolic number representations, analogous to a *scaffold*. The resultant early number competence (F3) comprises *tools* that are involved in arithmetic operations.

DD may be caused by different underlying deficits: analogous to a house construction, either the foundation itself is underdeveloped (F1), making it necessary to resort to domain-general skills as a scaffold (F2), which is less stable when lacking a proper foundation. But in other cases of DD, there are low-level domain-general skills such as working memory: despite an even foundation, an instable scaffold leads to an unsteady house. Third, if the link between non-symbolic and symbolic representations of number (F3) cannot be established, this is analogous to a craftsman using broken tools.

The empirical findings we outlined above are transferable to our three-factor account. Some children with DD show deficits hinting at a poor foundation (magnitude processing; F1), whereas other DD profiles rather accord with low scaffolding support (e.g., working memory or processing speed; F2). While this distinction is well established (Andersson and Östergren, 2012), our view on arithmetic development provides a novel approach to different DD patterns because it highlights the fact that math deficits may be present despite healthy domain-general and domain-specific skills. Crucially, early number competence (F3) is often subsumed under what we consider to be domain-specific skills (F1; as outlined above). By dissociating these two qualitatively different forms of cognition, future studies may succeed in disambiguating DD subtypes. In addition, contradictions between past studies are likely the cause of misconceptions of what a number sense is and what it is not, and distinct correlation patterns between ANS and formal (fact knowledge) compared with informal math (counting) yield empirical support showing a high face validity of this proposed concept (Libertus et al., 2013b). Thus, following Kolkman et al. (2013), domain-specific number sense (F1) primarily assists in establishing a successful mapping between magnitude and symbolic representations, i.e., prior to and during early arithmetic acquisition, for which there is empirical support (Inglis et al., 2011). These considerations are in line with the developmental model of number representation (Kucian and Kaufmann, 2009) introduced previously: while this descriptive model poses a solution for developmental changes observed with increasing arithmetic education, our three-factor account delivers a causal explanation for discrepant findings not only longitudinally (i.e., between different age groups) but also between conceptually different study designs within age groups. That transition appears to rely on domain-general skills (according to Namkung and Fuchs, 2016). Similarly, Hornung et al. (2014) postulate that early number competence in infants results from interacting basic quantity skills with domain-general abilities. We suggest that DD theories centered on domain-general deficits are primarily applicable to arithmetic acquisition and may therefore be considered valid especially in accounting for the high variability between age groups both in healthy and in clinical samples (see the discussion in Kaufmann et al., 2013).

Evidence for a third influencing factor (F3) comes from studies hinting at maleficent white matter tracts associated with poor math skills. Both interhemispheric fiber tracts between the IPL (representing the number sense, Cantlon et al., 2011) and intrahemispheric associations between IPL and angular gyrus could be verified (Klein et al., 2013).

Furthermore, the three-factor idea helps reconciling extreme positions of magnitude-based arithmetic vs. direct symbolic activation. The latter is assumed in the “encoding-complex model” (Campbell and Clark, 1988), which neglects domain-specific magnitude representations due to direct activation of numerosity based on parallel relative contributions of number representations. Thus, the foundation of early numerosity (F1) may seemingly become obsolete because studies often test early number competence (number acuity) with symbolic representations (F3). Indeed, recent findings hint at bidirectional correlations between number acuity (F3) and math skills (Lyons et al., 2012). While at first sight, this seems to contradict the assumed innateness of number sense processing (F1), it indeed strengthens our stance of a third independent factor of number acuity that was conceptualized as number sense in Lyons et al. (2012).

In addition, our account is sensitive to developmental shifts and therefore provides a high degree of flexibility. Future research should more clearly distinguish between the different concepts in order to differentiate between DD that is based on deficits with early numerosity from innate magnitude problems. Thus, Kaufmann et al. (2013) coined the expression *secondary DD* for low mathematical skills determined or even caused by low non-numerical cognitive skills. The matter is further complicated by findings that ANS (as a proxy for magnitude) and school-based math are reciprocally related (e.g., Nys et al., 2013; but see Zebian and Ansari, 2012) and that mathematical education impacts on ANS (Piazza et al., 2013; Lindskog et al., 2014). While such results question the assumed innateness of magnitude processing, they likewise provide implications for interventions. If numerosity (in terms of early number competence) turns out to be trainable, low-performing infants should be identified already before formal schooling and participate in specific training programs. Thus far, positive outcomes of such trainings on number acuity (e.g., Nys et al., 2013; Park and Brannon, 2013) could not be established beyond doubt (e.g., Zebian and Ansari, 2012; Obersteiner et al., 2013; see Szücs and Myers, 2017 for a review).

## Limitations

The articles on DD discussed in the present review are heterogeneous with respect to many aspects impacting on the study results. In the following, we will briefly outline the associated approaches and identify potential strengths and weaknesses:

### Design Considerations

#### Group Contrasts (Children With/Without DD)

These studies contrast children with DD and healthy age-matched controls with respect to various variables of interest (e.g., mathematical precursors; working memory; and language) and investigate the variance between both samples that each explains. While most efficient in terms of temporal and economic matters, such analyses provide little transferable information (small samples) and no basis to characterize putative causal relationships about developmental trajectories. For these purposes, cross-sectional and longitudinal studies are the means of choice.

#### Cross-Sectional vs. Longitudinal Studies

Both study types serve to reveal potential developmental processes. Cross-sectional studies offer a time-economic way to compare different developmental stages with each other but do not enable predictions about causal relations between possible precursor skills and later math achievements, in contrast to longitudinal studies.

### Methodological Considerations

#### Choice of Independent Variables

Another important issue concerns the choice of cognitive functions representing putative precursors and supportive skills for successful arithmetic development. Studies often either investigate domain-specific abilities (Chen and Li, 2014; Fazio et al., 2014) or domain-general skills (LeFevre et al., 2013; Bailey et al., 2014), even though controlling for one factor in the context of another or directly contrasting both (multidimensional approach, see Szücs, 2016) may deliver a more comprehensive insight (Aunola et al., 2004). In addition, studies concentrating on one category (i.e., specific or general) often fail to take into account a sufficient amount of associated factors.

#### Choice of Dependent Variables

There is no unitary operationalization of math proficiency or achievement, nor are age and school-based development adequately accounted for. As a result, heterogeneous constructs such as number system knowledge (Sowinski et al., 2015), timed math (Sasanguie et al., 2013), mental calculation(Reeve et al., 2015), standardized math test scores (Chang et al., 2015), or arithmetic fluency (LeFevre et al., 2013) exist for the same overall latent variable termed math proficiency.

### Considerations on Sample Criteria

#### Developmental Trajectories

Irrespective of study design, the classification and comparability of participant sub-groups impact on the associated gain of knowledge. In arithmetic research, longitudinal studies often attend healthy children during the transition from kindergarten to preschool or primary school, thus allowing predictions about regular mathematical proficiency (e.g., VanDerHeyden et al., 2006; Lembke and Foegen, 2009; Siegler et al., 2012). However, the informational value in terms of developmental trajectories of mathematical disorders is limited. Consequently, longitudinal studies on children with low initial number processing abilities (number sense) and potential struggles arising with symbolic representations thereof (transcoding) are more suitable. For this, screening instruments are required that test preschoolers on non-symbolic number processing (e.g., mental number line or non-symbolic quantity estimation tests).

#### DD Definitions and Diagnostic (Cut-Off) Criteria

As with math proficiency, the criteria required to sort children into DD (sub)groups are equally inconsistent (see Murphy et al., 2007 for a review). Some studies use the term “persistent mathematical difficulties” to dissociate putative genuine DD from mild and potentially transitional numerical difficulties (Morgan et al., 2016), whereas others collapse over these categories (e.g., Murphy et al., 2007). Furthermore, studies seldom take into account ICD-10 diagnostic criteria for DD, and the associated cut-off criteria are commonly weakened, ranging from the 10th to the 35th percentile (Mazzocco and Thompson, 2005). Thus, qualitative differences between persistent and transient arithmetic weaknesses (Mazzocco and Myers, 2003) impede the comparability between study samples that are based on moderate (e.g., Jordan et al., 2003; Geary, 2004) or low math achievement scores (Mazzocco et al., 2011). Besides, reporting comorbid deficits is no established practice, even though these pose additional and fundamental developmental challenges (Szücs, 2016). In addition, concerns have been raised in the past due to the conceptual overlap between mathematical tests with IQ subtests. As a recent advance, the discrepancy criterion was abolished in the United States in 2013 (Schulte-Körne, 2014). DD research mainly relies on convenience samples (often school samples) where standardized IQ indices are not reported at all, or otherwise intelligence level was included as a domain-general regressor. In this respect, weakening the diagnostic criteria as frequently done in DD studies may be advantageous. However, lowering the required discrepancy between participants’ math test score and their age-based reference group may be more problematic. Assuming that math skills are dimensionally distributed, this approach may falsely sort healthy low performers into the group of DD patients. This matter is further complicated by the ambiguous definition of DD and potential subgroups.

### Considerations on Selected Studies

The present article should not be misunderstood as a systematic or exhaustive review nor does it make the claim to cover all relevant open questions about DD. The choice of studies is selective and we may not have covered all relevant viewpoints or theories on the related issues. Rather, we wish to point to one essential gap in the approach to research in this field. By drawing attention to the ambiguous conceptualization of “the number sense,” we hope to initiate a finer distinction between the discussed abilities/skills in shaping arithmetic sophistication. This may provide new ways of interpreting study results and help reconciling discrepant findings.

In sum, the available studies on DD and math development are confounded with many influential factors. This article served to sensitize researchers in this matter by contrasting evidence and standpoints in the literature from many angles. We complemented these considerations by introducing a novel approach that equally applies to the interpretation of contradictory study results as to the classification of DD subtypes. Thereby, we wished to close this gap and answer some of the questions that follow when looking at individual study results.

## Outlook

In order to differentiate between genuine DD and low math abilities, individual developmental trajectories should be considered in the context of various contributing skills. This idea is pressing given the broad field of domain-general and domain-specific precursors that each demonstrates interindividual differences. Disentangling low but healthy math performance from clinically relevant and persistent DD is essential and requires multilevel diagnostic instruments. These in turn depend on the identification of unique precursors of DD that should be screened early on in preschool. For that purpose, future studies are needed that address math development prior to formal mathematical education. As for now, the majority of studies examined school-aged samples, i.e., after having acquired the basic concepts of arithmetic. So far, findings on early number competence (before kindergarten) are still lacking (Morgan et al., 2016). Such studies would help to further disentangle innate abilities (F1) from acquired numerical skills (F3). Furthermore, contradictions between existing studies can possibly be reconciled in a meta-analysis when introducing our three-factor approach.

## Author Contributions

JS wrote the preliminary draft. JS and FP revised the manuscript and read and approved the final manuscript.

## Conflict of Interest Statement

The authors declare that the research was conducted in the absence of any commercial or financial relationships that could be construed as a potential conflict of interest.
